# The arc of discovery, from the description of cystic fibrosis to effective treatments

**DOI:** 10.1172/JCI186231

**Published:** 2024-10-01

**Authors:** Michael J. Welsh

**Affiliations:** Departments of Internal Medicine, Molecular Physiology and Biophysics, Neurology, and Neurosurgery, Pappajohn Biomedical Institute, Howard Hughes Medical Institute, Roy J. and Lucille A. Carver College of Medicine, University of Iowa, Iowa City, Iowa, USA.

The story of investigation and discovery that led to effective treatments for cystic fibrosis (CF) might be considered a model for pursuing a genetic disease and developing successful therapies. It is apt to recount this story as we mark the 100th anniversary of the *JCI* because of the journal’s tradition of describing the mechanistic bases of disease and an understanding of how they might be altered to impact human health.

Let me begin by giving credit to the trainees, physicians, scientists, and caregivers around the world and in my own lab who propelled this journey. I also thank the people with CF who participated in the research and inspired us. The work was enabled by the Cystic Fibrosis Foundation (CFF), the NIH, the Howard Hughes Medical Institute (HHMI), and many donors.

## Identification of the clinical disease and initial therapies

For me, as for many physician-scientists, the story began with a patient. As a third-year medical student at the University of Iowa, I entered a clinic room and saw a young girl coughing and struggling to breathe. Her prognosis was dismal, and the lack of effective treatments was disheartening. That girl remains deeply embedded in my memory.

Decades earlier, Dorothy Andersen, a pathologist at Babies Hospital at Columbia-Presbyterian, identified fluid-filled cysts surrounded by scars in the pancreas in autopsies of children originally thought to have celiac disease. In 1938, she named the disease “cystic fibrosis of the pancreas” (1). She and others rapidly recognized the severe bronchiectasis and autosomal recessive inheritance that are associated with CF.

A breakthrough came after the New York heat wave of 1948, when children with CF were brought to the hospital with severe dehydration and heat prostration. Paul di Sant’Agnese discovered that people with CF lose excessive salt into sweat, which led to a diagnostic test — the sweat Cl^–^ test — that became the cornerstone of CF diagnosis (2).

Increased diagnostic accuracy and recognition of the disease further revealed that CF affects multiple organs. In addition to pancreas and lung destruction, it causes liver disease, meconium ileus, male sterility, and CF-related diabetes. However, it is the lung disease, with recalcitrant bacterial infections, profuse inflammation, and copious mucus production, that causes most of the morbidity and mortality. In the 1960s, few children survived beyond their teenage years.

Progress for people with CF came when physicians, scientists, and caregivers began newborn screening programs and established CF clinical centers and a CFF Therapeutics Development Network. In addition, they developed better therapies, including pancreatic enzyme replacement, nutritional support, chest percussion, aggressive antibiotic therapy (including inhaled antibiotics), inhaled DNase, inhaled hypertonic saline, and lung transplantation. By the end of the 1980s median survival had increased to approximately 18 years.

## Insights into the pathophysiology

From the 1960s to 1980s, the quest to explain how this genetic disease caused the diverse manifestations accelerated. A 1962 *JCI* paper reported abnormal mucoproteins in CF urine as an early attempt to identify the molecular nature of the disease (3).

A fundamental discovery came in 1983 when Paul Quinton reported that, in CF, the sweat gland duct had an abnormally low Cl^–^ permeability — hence, impaired salt reabsorption and a high sweat Cl^–^ concentration (4). Shortly thereafter, a *JCI* paper indicated that CF airways also had a reduced Cl^–^ permeability (5). We then localized the defect to a loss of Cl^–^ conductance in the airway epithelial apical membrane (6).

With these findings, and with subsequent advances in epithelial culture techniques and physiological studies, a unifying hypothesis emerged — CF disrupts epithelial anion conductance.

## Identification of the *CFTR* gene and discovery of CFTR function

In 1989, our lab huddled around our fax machine as it rolled out Lap-Chee Tsui, Francis Collins, and their colleagues’ paper identifying the gene that is mutated in CF — cystic fibrosis transmembrane conductance regulator (*CFTR*) (7). The *CFTR* gene was one of the first positional cloning successes, a remarkable accomplishment given the absence of established chromosome and genome maps. As we pored over the paper, we wondered, “What does CFTR do?”

Within a year, we and others reported that expressing wild-type CFTR corrected defective Cl^–^ transport, whereas expressing *CFTR* bearing the common CF-associated mutation, *ΔF508* (now *F508del*), did not (8, 9). However, the predicted primary structure of CFTR left the field puzzled about its function. Because CFTR belongs to the large ATP-binding cassette (ABC) transporter family, the field assumed that, like other ABC transporters, CFTR performed some form of active transport that somehow regulated Cl^–^ permeability, hence its name as a “transmembrane conductance regulator.”

Nevertheless, we proposed the simple hypothesis that CFTR itself is an anion channel. We tested that hypothesis, and I remember our excitement at the results, including Matt Anderson running out of our patch-clamp room yelling, “I can’t believe it worked!” Thus, CFTR is unique among ABC transporters; it is a channel that provides a path for passive anion flow across membranes (10).

Subsequent studies probed the mechanisms by which anions permeate through the channel pore, ways that cAMP-dependent phosphorylation activates the channel, and how ATP hydrolysis and adenylate kinase activity control channel gating. The results and insights from those studies provided a foundation for modifying CFTR function therapeutically.

The discoveries of CFTR and its function marked a major inflection point. They tied together the previous understanding of the genetics and the physiology. And looking forward, they allowed more precise diagnostic testing, opened new opportunities to understand the disease, and suggested that measuring ion channel function could provide a critical assay for discovering CFTR-targeted therapeutics.

## Disruption of CFTR function by mutations

With the gene in hand, investigators raced to identify disease-causing mutations. *CFTR-ΔF508* is the most common, accounting for approximately 85% of mutations in the United States. The presence of a single allele accounting for the majority of cases is what enabled successful mapping and identification of the gene. With sequencing methods available now, more than 2000 *CFTR* variants have been reported, and more than 700 disrupt function.

This complexity became manageable and later actionable by grouping disease-causing mutations into different classes (11). Class 1 mutations interfere with production of CFTR. Class 2 mutations (e.g., *CFTR-ΔF508*) disrupt the 3D structure of CFTR and prevent its processing and delivery to the apical membrane. Class 3 mutations (e.g., *CFTR-G551D*) impair channel gating, reducing the open time. Class 4 mutations form channels with reduced anion conduction. Class 5 mutations produce insufficient amounts of protein. Class 6 mutations have reduced stability in the cell membrane. Some mutants exhibit more than one defect; for example, *CFTR-ΔF508* is both misprocessed and has decreased opening (Classes 2 and 3).

This classification scheme and an understanding of how mutations disrupt CFTR function were critical advances; they provided a roadmap for developing mutation-specific therapies that target the fundamental defects in CFTR, i.e., precision medicine.

## Rescue of CFTR mutants and effective therapies

An intense effort to improve mutant CFTR function ensued. Knowing that temperature sensitivity was used to study mutant proteins in lower organisms, we reduced the incubation temperature and were ecstatic when we saw mature CFTR-ΔF508 on a gel; it escaped the endoplasmic reticulum and trafficked to the cell surface where it retained substantial, albeit reduced, activity (12). That result demonstrated that mutant CFTR could be rescued and suggested that a chemical might substitute for reduced temperature. Identification of second-site suppressors supported this idea. Those findings ignited efforts to identify small molecules that correct specific CFTR defects.

Critical progress came when Bob Beall and the CFF funded Aurora Biosciences to use their high-throughput screening assays to identify compounds that could become novel therapeutics, at a scale not feasible in academia. Vertex Pharmaceuticals acquired Aurora and continued the program with the development of what became ivacaftor, a chemical that increases channel opening of the Class 3 mutant CFTR-G551D. Ivacaftor dramatically improved lung function in people with CF bearing a *CFTR-G551D* mutation. Continued efforts ultimately yielded a highly effective three-drug combination — elexacaftor, tezacaftor, and ivacaftor (ETI) — with elexacaftor and tezacaftor “correcting” CFTR-ΔF508 misprocessing, and ivacaftor “potentiating” channel opening (13, 14). These drugs highlight the power of compound screens that target very specific modalities.

The results were extraordinary. ETI markedly improved lung function, even in people with one *CFTR-ΔF508* mutation. Pulmonary exacerbations fell, respiratory symptoms decreased, and body weight increased. These benefits extended beyond *CFTR-ΔF508* to several other mutations, thus initiating in vitro testing of modulators on patient-derived cells and CFTR mutants to predict clinical efficacy for people with rare CFTR variants. Within 2 years, the number of lung transplants for the sickest patients plunged by nearly 80%. For me, more striking than numbers have been personal stories of people with CF competing in a state cross country race, getting married, having children, and planning for retirement.

## The horizon

The coach of my son’s winning cross country team often said, “We’re not done yet.” The same is true in CF, with a focus on developing treatments for people who do not have modulator-responsive mutations, have limited benefit from current modulators, or have adverse effects. Genetic approaches offer an appealing option. Within 5 years after *CFTR* identification, we and others demonstrated the feasibility of *CFTR* gene transfer to airways in people with CF. But “gene transfer” was too inefficient for “gene therapy” (15, 16). Undaunted, we and the field identified barriers to gene transfer, and that work, together with exciting opportunities presented by new gene transfer and editing approaches suggest that a genetic treatment will ultimately be successful for CF. Other approaches are attempting to correct CFTR in specific mutation classes or to develop mutation-agnostic treatments. Another strategy is developing better modulators, perhaps guided by recent beautiful CFTR structural studies and the realization that modulators may activate some non-CFTR channels (17).

Improving therapies for all people with CF will benefit from further understanding CF pathophysiology. Lack of animal models have bottlenecked research; *Cftr*-mutated mice do not develop CF lung disease. More recent development and studies of pigs, ferrets, rats, and sheep with *CFTR* mutations are revealing how the loss of CFTR activity disrupts host defenses in lungs and alters function in other organs (18, 19). Understanding impaired function in other organs is increasingly important because, whereas modulators reach CFTR throughout the body, agents that target only the epithelial cells lining respiratory airways do not affect the CF metabolic abnormalities of multiple other organs (20).

## Closing

It has been an incredible privilege to follow the arc of discovery, from caring for a young patient in the clinic, through research uncovering the genetic and pathophysiologic defects, and back to people with highly effective treatments that have changed their lives. As with any scientific and clinical journey, new questions and challenges will arise. But we can now see more clearly, and we’re not done yet.

## Version 1 10/01/2024

Electronic publication
